# Validity of International Classification of Diseases (ICD) coding for dengue infections in hospital discharge records in Malaysia

**DOI:** 10.1186/s12913-018-3104-z

**Published:** 2018-04-20

**Authors:** Yuan-Liang Woon, Keng-Yee Lee, Siti Fatimah Zahra Mohd Anuar, Pik-Pin Goh, Teck-Onn Lim

**Affiliations:** 1 National Clinical Research Centre, Ministry of Health Malaysia, Level 3, Dermatology Block, Kuala Lumpur Hospital, Jalan Pahang, 50586 Kuala Lumpur, Malaysia; 2 Clin Research Sdn Bhd, D7-3-1, Block D7, Pusat Perdagangan Dana 1, Jalan PJU 1A/46, PJU 1A, 47301 Petaling Jaya, Selangor Malaysia

**Keywords:** Dengue, Health services, Validation, International classification of diseases, Diagnosis, Malaysia

## Abstract

**Background:**

Hospitalization due to dengue illness is an important measure of dengue morbidity. However, limited studies are based on administrative database because the validity of the diagnosis codes is unknown. We validated the International Classification of Diseases, 10th revision (ICD) diagnosis coding for dengue infections in the Malaysian Ministry of Health’s (MOH) hospital discharge database.

**Methods:**

This validation study involves retrospective review of available hospital discharge records and hand-search medical records for years 2010 and 2013. We randomly selected 3219 hospital discharge records coded with dengue and non-dengue infections as their discharge diagnoses from the national hospital discharge database. We then randomly sampled 216 and 144 records for patients with and without codes for dengue respectively, in keeping with their relative frequency in the MOH database, for chart review. The ICD codes for dengue were validated against lab–based diagnostic standard (NS1 or IgM).

**Results:**

The ICD-10-CM codes for dengue had a sensitivity of 94%, modest specificity of 83%, positive predictive value of 87% and negative predictive value 92%. These results were stable between 2010 and 2013. However, its specificity decreased substantially when patients manifested with bleeding or low platelet count.

**Conclusion:**

The diagnostic performance of the ICD codes for dengue in the MOH’s hospital discharge database is adequate for use in health services research on dengue.

**Electronic supplementary material:**

The online version of this article (10.1186/s12913-018-3104-z) contains supplementary material, which is available to authorized users.

## Background

Dengue has become a global public health concern. Epidemiologic measures of the burden of dengue such as its incidence and prevalence are important for policy-making and monitoring the progress of disease control. WHO reported the global incidence of dengue has increased 30-fold in the past 50 years and estimated some 50 to 100 million new infections occurred annually [1], causing about 20,000 deaths [2]. These estimates are largely based on dengue notification data reported to national surveillance systems, which are widely used as a proxy measure of dengue incidence [3–7].

However, for dengue where the majority of infected individuals are asymptomatic and may suffer no or little adverse health consequences, estimates of incidence and prevalence are measures of disease frequency rather than measures of the disease burden. Case fatality rate of dengue remains low with an average of 2.4 deaths over 100,000 notified case in Malaysia [8], but symptomatic dengue infection is significantly associated with considerable morbidity. Hospitalization due to an acute illness like symptomatic dengue infection is an important measure of morbidity. Dengue hospitalization is a critical driver of economic cost of the illness to society, especially in a country endemic of dengue. Apart from the medical costs, hospitalization also leads to loss in economic productivity arising of sick workers taking sick leaves or parents taking time off to care for their sick children. Nonetheless, health services utilization associated with dengue illness, and hospitalization in particular, remains poorly characterized, though there have been many hospital based, mostly single-center, studies on dengue [9–17]. Few studies have make use of administrative data which are available from hospital discharge and health insurance claims databases [18] in part because the validity of discharge diagnoses codes is uncertain. We therefore undertook this study to evaluate the accuracy of discharge diagnoses coded according to the International Classification of Diseases, Tenth Revision, Clinical Modification (ICD-10-CM) codes for dengue. Validation of these codes is necessary to promote the wider use of hospital discharge and insurance claims data for health services research on dengue.

## Methods

This validation study extracted data from the available hospital discharge records and retrospective review of medical records. The Medical and Research Ethics Committee approved the study (NMRR-15-452-25,624).

### Hospital discharge records

The Health Informatics Centre (HIC) of the Ministry of Health (MOH) Malaysia maintains a data warehouse containing data on hospital discharges from both public and private hospitals in the country. Discharge diagnoses are coded according to the International Classification of Diseases, Tenth Revision, Clinical Modification (ICD-10-CM). Using this database, we randomly extracted 3219 discharge records across all age groups from seven hospitals for the years 2010 and 2013. These are tertiary hospitals located in Peninsular Malaysia. The hospitals were conveniently sampled according to their geographical locations. Three of them are located at the Central (Kuala Lumpur Hospital, Tengku Ampuan Rahimah Hospital and Subang Jaya Medical Centre); two are located at the North (Seberang Jaya Hospital and Sultanah Bahiyah Hospital); one located at the South (Sultanah Aminah Hospital); and another one located at the East Coast of Peninsular Malaysia (Tengku Ampuan Afzan Hospital). Records coded with dengue include A90 for dengue fever [classical dengue] and A91 dengue haemorrhagic fever. Codes for records without dengue such as B34.9, A83, R50, A92 to A99 were categorized as non-dengue. Refer to Additional file 1 for detailed descriptions of these ICD-10-CM codes. We deliberately restricted selection of non-dengue records to these codes (B34.9, A83, R50, A92 to A99) because these discharges that are most likely a priori to be misclassified as false-positive for dengue.

### Review of medical records and laboratory data

We abstracted data from medical and laboratory records for these 3219 discharge records from the seven hospitals. We obtained information on patients’ demographics, dates of admission and discharge, disease severity and laboratory results for dengue non-structural protein 1 (NS1) antigen immunoassay and dengue immunoglobulins M (IgM) assay. Records of patients coded with dengue constituted 2.9% of HIC database while records coded with either B34.9, A83, R50, or A92 to A99 constituted 2.0%. Estimates of PPV and NPV are sensitive to the prevalence of the dengue. We therefore randomly sampled 216 records for patients with codes for dengue and 144 records without dengue codes, in keeping with their relative frequency in the database. Only records with complete medical and laboratory data for evaluation are included in the sampling.

### Diagnostic performance of ICD-10-CM codes for dengue

We compared discharge diagnoses ICD codes for dengue against the standard diagnostic tests based on dengue NS1 and IgM. Both NS1 and IgM might not identify all dengue cases presenting to the hospitals but they are sufficiently accurate to be recommended for routine diagnostic use [19, 20]. NS1 and IgM are the only two diagnostic tests routinely available in almost all hospitals in Malaysia. Public health surveillance in Malaysia is also based on these two tests to ascertain occurrence of dengue in the population. More accurate diagnostic methods such as plaque reduction neutralisation test, polymerase chain replication-based assay or virus isolation [19] are either non-routinely performed or not available at all hospitals in Malaysia. For this study we defined a record of having a true diagnosis of dengue if showed a positive result for NS1 and/or IgM. We then calculated the sensitivity, specificity, positive predictive value (PPV), and negative predictive value (NPV) of ICD-10-CM codes for dengue against this lab–based diagnostic standards.

### Statistical methods

The background demography, clinical manifestations and clinical outcome of sampled cases were described. All categorical variables were presented as frequencies and percentages; while continuous variables were expressed as means with standard deviation (SD) or medians with interquartile ranges (IQR). The background characteristics between patients with and without ICD-10-CM codes for dengue were examined with Chi-square test for categorical variables, and Student’s T-test or Mann-Whitney U tests for numerical variables. The two-sided statistical significance level was set at 0.05. We also estimated the binomial exact 95% confidence intervals (CIs) for the sensitivity, specificity, PPV and NPV of ICD-10-CM codes for dengue.

## Results

Table 1 shows the characteristics of the patients with and without ICD-10-CM codes for dengue whom were included in this study. Their mean age were 37 and 28 years respectively. About half the patients were diagnosed in year 2010, and the other half in 2013. Bleeding, visceral signs, low platelet count, raised blood hematocrit were more commonly present among patients with ICD-10-CM codes for dengue. Organ impairment however were found equally between the two groups. Table 2 summarizes the number of cases with positive result for NS1 and/or IgM among patients with and without ICD-10-CM codes for dengue. 87% of those with the dengue codes had positive laboratory results (NS1 or IgM) for dengue while only 8% of those without dengue codes had positive results.

**Table 1:** Characteristics of patients with and without ICD-10-CM codes for dengue

	Patients with ICD-10-CM codes for Dengue (A90, A91)	Patients without ICD-10-CM codes for Dengue (B34.9, A83, R50, A92 to A99)	Statistical test	*P*-value
	*N* = 216	*N* = 144		
Year of diagnosis				
2010, *n* (%)	86 (40)	73 (51)	Chi-square test	0.042
2013, *n* (%)	130 (60)	71 (49)		
Median Length of Stay (IQR), day	3 (2)	2.5 (2)	Mann-Whitney U test	< 0.001
Age, year				
Mean (SD)	37 (15)	28 (18)	Student’s T-test	< 0.001
Minimum	3	1		
Maximum	79	74		
Gender				
Male, *n* (%)	118 (55)	76 (53)	Chi-square test	0.730
Female, *n* (%)	98 (44)	68 (47)		
Ethnicity				
Malay, *n* (%)	115 (53)	58 (40)	Chi-square test	0.015
Chinese, *n* (%)	50 (23)	55 (38)		
Indian, *n* (%)	30 (14)	21 (15)		
Others, *n* (%)	21 (10)	10 (7)		
Clinical manifestation				
Bleeding, *n* (%)	44 (20)	9 (6)	Chi-square test	< 0.001
Visceral signs, *n* (%)	105 (49)	22 (15)	Chi-square test	< 0.001
Low platelet count, *n* (%)	95 (44)	7 (5)	Chi-square test	< 0.001
Raised hematocrit, *n* (%)	16 (7)	0 (0)	Chi-square test	0.001
Organ impairment, *n* (%)	15 (7)	9 (6)	Chi-square test	0.796
Dead at discharge, *n* (%)	1 (0.5)	0 (0.0)	Chi-square test	0.414

**Table 2:** Comparison of NS1 and/or IgM Result between Patients with and without ICD-10-CM Codes for Dengue

Dengue Cases	Positive NS1/Ig M	Negative NS1/Ig M	Total
	Frequency, *n*	Frequency, *n*	
Patients with ICD-10-CM codes for Dengue	188	28	216
Patients without ICD-10-CM codes for Dengue	12	132	144
Total	200	160	360

Table 3 shows the diagnostic performance of the ICD-10-CM codes for dengue. The sensitivity was 94% (188/ 200) and specificity 83% (132/160), giving an overall accuracy of 88.9% (320/ 360). The positive Likelihood ratio (LR) was 4.7 and negative LR 0.075. This provides adequate evidence for the diagnostic validity of ICD dengue codes. Its PPV was 87% (188/ 216) and NPV 92% (132/ 144). The diagnostic performance of dengue codes did not change between 2010 and 2013 (Table 3). Neither did it differ between the sexes. However, the specificity of ICD-10-CM codes for dengue decreased substantially when the patients presented with bleeding manifestations or low platelet count.

**Table 3:** Diagnostic performance of ICD-10-CM codes for Dengue validated against positive NS1 or IgM lab results as diagnostic standards

	*N*	Sensitivity, % (95% CI)	Specificity, % (95% CI)	Positive predictive value, % (95% CI)	Negative predictive value, % (95% CI)
Overall	360	94	83	87	92
		(92, 97)	(79, 87)	(83, 90)	(89, 95)
Year 2010	159	91	88	90	89
		(86, 95)	(82, 93)	(83, 94)	(83, 94)
Year 2013	201	97	78	85	96
		(94, 99)	(72, 84)	(79, 90)	(92, 98)
Male	194	94	81	86	92
		(90, 97)	(75, 86)	(80, 91)	(87, 95)
Female	166	95	84	88	93
		(91, 98)	(77, 89)	(82, 92)	(88, 97)
Bleeding	53	95	58	89	78
		(84, 99)	(44, 72)	(77, 96)	(64, 88)
No bleeding	307	94	85	87	93
		(91, 96)	(81, 89)	(83, 91)	(90, 96)
Low platelet count < 50	102	98	31	88	77
		(93, 100)	(23, 41)	(80, 94)	(68, 85)
Platelet count ≥ 50	258	92	88	86	93
		(88, 95)	(83, 92)	(81, 90)	(89, 96)

## Discussion

Administrative data such as hospital discharge and health insurance claims databases have infrequently been used for health services research on dengue despite their considerable strengths which included nationally representative sample to allow generalizability, larger sample size and low cost. The main disadvantage of administrative data is uncertain validity of the ICD codes used to identify patients with dengue. This study was undertaken to address this weakness and is the first to investigate the validity of ICD-10-CM codes for dengue. Overall we demonstrated adequate diagnostic evidence to support the use of ICD-10-CM codes in identifying hospitalized patients with positive NS1 or IgM test for dengue.

The sensitivity of ICD-10-CM codes for dengue is high (94%), but its specificity is more modest at 83% (17% false positive ratio). The lower specificity could have two explanations. Firstly, despite advances in dengue diagnostic tests [19], the diagnosis of dengue in current practice is still clinically based rather than entirely dictated by positive NS1 or IgM lab results [20]. This is further supported by our findings of lower specificities when patients manifested with bleeding or low platelet count, both commonly associated with dengue. In other words, patients are more likely to be clinically diagnosed of having dengue even when their NS1 or IgM tests were negative. Secondly, our records selection process also contributed to this modest specificity result. We have deliberately restricted selection of records without ICD codes for dengue to B34.9, A83, R50, A92 to A99 codes because they are most likely a priori to be misclassified as false-positive, thus lowering the specificity estimates. If we would have randomly selected all records without ICD codes for dengue as controls, we would then find nearly all to be true negative given the low prevalence (< 3%) of dengue among all hospitalized subjects, thus inflating the estimate of specificity.

Our results have several implications. For the use of administrative data in descriptive studies to estimate the incidence or prevalence of patients hospitalized for dengue illness, the high sensitivity of A90–91 codes ensures that most of the dengue patients will be identified. However, its moderate specificity (83%) will inflate the estimate of the true frequency of dengue in the population, with high number of false positives. Even moderate loss in specificity can lead to substantial over-estimation especially when the incidence or prevalence of the condition under investigation is low. Our finding that the validity of the ICD-10-CM codes has been stable over time is reassuring for studies on the secular trend in dengue incidence or prevalence.

For analytic studies, the ICD-10-CM codes can be used to sample cases of dengue and non-dengue controls to investigate outcome of subjects’ exposure to dengue. Assuming non-differential misclassification (ie, classification by ICD-10-CM codes is independent of the outcome of interest) and assuming having dengue increases risk of the outcome, the modest specificity (83%) of A90–91 codes will cause cases to have more false positive subjects among them to dilute the risk estimate of the case group, while the high sensitivity (94%) will minimize the number of false negative subjects in the control group, thus lessening its risk inflation. The end result will be a biased estimate of the relative risk towards null.

### Limitations of study

As this study involved only seven hospitals, the findings cannot be generalized to all hospitals in Malaysia. In addition, NS1 and/or IgM were used as the diagnostic standards to validate the ICD-10-CM codes for dengue. In this case, both NS1 and IgM are not perfect tests, so some of the dengue cases may be missed. Nevertheless, both NS1 and IgM are the only diagnostic tests that are available in almost all hospitals in Malaysia. Lastly, as this study involved only data from 2010 and 2013, the observed trend of ICD-10-CM validity may not be entirely true for the data of other years.

## Conclusion

We conclude that the hospital discharge database maintained by the Ministry of Health (MOH) Malaysia is potentially a useful data resource to support health services research for dengue in Malaysia. The adequate diagnostic performance of the ICD-10-CM coding in the national hospital discharge database in identifying patients with dengue makes it particularly acceptable for use in research on dengue.

## Additional file

Additional file 1: ICD-10-CM Diagnosis Codes. (DOCX 13 kb)

## Abbreviations

ICD-10: International Classification of Diseases, 10th revision
IgM: Immunoglobulins M assay
IQR: Interquartile range
MOH: Ministry of Health
NPV: Negative predictive value
NS1: Non-structural protein 1 antigen
PCR: Polymerase Chain Reaction
PPV: Positive predictive value
WHO: World Health Organization
