# Supplementary material for: Validity of International Classification of Diseases (ICD) coding for dengue infections in hospital discharge records in Malaysia
Source: BMC Health Serv Res. 2018 Apr 20;18:292. doi: 10.1186/s12913-018-3104-z (PMC5910617; doi:10.1186/s12913-018-3104-z)
Supplement: Supplementary file 1 — ICD-10-CM Diagnosis Codes. (DOCX 13 kb) [file 12913_2018_3104_MOESM1_ESM.docx]

# Additional file 1: ICD-10-CM Diagnosis Codes

*Dengue: any of these 2 codes:*

| A90 | Dengue fever [classical dengue] |
| --- | --- |
| A91 | Dengue haemorrhagic fever |

*Non-Dengue: any of these codes:*

| B34.9 | Viral infection, unspecified |
| --- | --- |
| A83 | Mosquito-borne viral encephalitis |
| R50 | Fever of unknown origin |
| A92 | Other mosquito-borne viral fevers |
| A92.0 | Chikungunya virus disease |
| A92.1 | O'nyong-nyong fever |
| A92.2 | Venezuelan equine fever |
| A92.3 | West Nile fever |
| A92.4 | Rift Valley fever |
| A92.8 | Other specified mosquito-borne viral fevers |
| A92.9 | Mosquito-borne viral fever, unspecified |
| A93 | Other arthropod-borne viral fevers, not elsewhere classified |
| A93.0 | Oropouche virus disease |
| A93.1 | Sandfly fever |
| A93.2 | Colorado tick fever |
| A93.8 | Other specified arthropod-borne viral fevers |
| A94 | Unspecified arthropod-borne viral fever |
| A95 | Yellow fever |
| A95.0 | Sylvatic yellow fever |
| A95.1 | Urban yellow fever |
| A95.9 | Yellow fever, unspecified |
| A96 | Arenaviral haemorrhagic fever |
| A96.0 | Junin haemorrhagic fever |
| A96.1 | Machupo haemorrhagic fever |
| A96.2 | Lassa fever |
| A96.8 | Other arenaviral haemorrhagic fevers |
| A96.9 | Arenaviral haemorrhagic fever, unspecified |
| A98 | Other viral haemorrhagic fevers, not elsewhere classified |
| A98.0 | Crimean-Congo haemorrhagic fever |
| A98.1 | Omsk haemorrhagic fever |
| A98.2 | Kyasanur Forest disease |
| A98.3 | Marburg virus disease |
| A98.4 | Ebola virus disease |
| A98.5 | Haemorrhagic fever with renal syndrome |
| A98.8 | Other specified viral haemorrhagic fevers |
| A99 | Unspecified viral haemorrhagic fever |
